# Escalating SARS-CoV-2 specific humoral immune response in rheumatoid arthritis patients and healthy controls

**DOI:** 10.3389/fimmu.2024.1397052

**Published:** 2024-06-07

**Authors:** Dora Nemeth, Hajnalka Vago, Laszlo Tothfalusi, Zsuzsanna Ulakcsai, David Becker, Zsofia Szabo, Bernadett Rojkovich, Lilla Gunkl-Toth, Bela Merkely, Gyorgy Nagy

**Affiliations:** ^1^ Department of Rheumatology and Clinical Immunology, Semmelweis University, Budapest, Hungary; ^2^ Department of Internal Medicine and Oncology, Semmelweis University, Budapest, Hungary; ^3^ Department of Genetics, Cell- and Immunobiology, Semmelweis University, Budapest, Hungary; ^4^ Heart and Vascular Center, Semmelweis University, Budapest, Hungary; ^5^ Department of Sports Medicine, Semmelweis University, Budapest, Hungary; ^6^ Department of Pharmacodynamics, Semmelweis University, Budapest, Hungary; ^7^ Department of Laboratory Medicine, Semmelweis University, Budapest, Hungary; ^8^ Buda Hospital of the Hospitaller Order of Saint John of God, Budapest, Hungary; ^9^ Department of Pharmacology and Pharmacotherapy, Medical School, University of Pécs, Pécs, Hungary; ^10^ Chronic Pain Research Group, Hungarian Research Network - University of Pécs (HUN-REN-PTE), Pécs, Hungary

**Keywords:** SARS-CoV-2, humoral immune response, cellular immune response, longitudinal study, immunosuppression

## Abstract

**Background:**

Immunocompromised patients are at particular risk of Severe Acute Respiratory Syndrome Corona Virus 2 (SARS-CoV-2) infection and previous findings suggest that the infection or vaccination induced immune response decreases over time. Our main goal was to investigate the SARS-CoV-2-specific immune response in rheumatoid arthritis patients and healthy controls over prolonged time.

**Methods:**

The SARS-CoV-2-specific humoral immune response was measured by Elecsys Anti-SARS-CoV-2 Spike (S) immunoassay, and antibodies against SARS-CoV-2 nucleocapsid protein (NCP) were also evaluated by Euroimmun enzyme-linked immunosorbent assay (ELISA) test. The SARS-CoV-2-specific T-cell response was detected by an IFN- γ release assay.

**Results:**

We prospectively enrolled 84 patients diagnosed with rheumatoid arthritis (RA) and 43 healthy controls in our longitudinal study. Our findings demonstrate that RA patients had significantly lower anti-S antibody response and reduced SARS-CoV-2-specific T-cell response compared to healthy controls (p<0.01 for healthy controls, p<0.001 for RA patients). Furthermore, our results present evidence of a notable increase in the SARS-CoV-2-specific humoral immune response during the follow-up period in both study groups (p<0.05 for healthy volunteers, p<0.0001 for RA patients, rank-sum test). Participants who were vaccinated against Coronavirus disease-19 (COVID-19) during the interim period had 2.72 (CI 95%: 1.25–5.95, p<0.05) times higher anti-S levels compared to those who were not vaccinated during this period. Additionally, individuals with a confirmed SARS-CoV-2 infection exhibited 2.1 times higher (CI 95%: 1.31–3.37, p<0.01) anti-S levels compared to those who were not infected during the interim period. It is worth noting that patients treated with targeted therapy had 52% (CI 95%: 0.25–0.94, p<0.05) lower anti-S levels compared to matched patients who did not receive targeted therapy. Concerning the SARS-CoV-2-specific T-cell response, our findings revealed that its level had not changed substantially in the study groups.

**Conclusion:**

Our present data revealed that the level of SARS-CoV-2-specific humoral immune response is actually higher, and the SARS-CoV-2-specific T-cell response remained at the same level over time in both study groups. This heightened humoral response, the nearly permanent SARS-CoV-2-specific T-cell response and the coexistence of different SARS-CoV-2 variants within the population, might be contributing to the decline in severe COVID-19 cases.

## 1 Introduction

The emergence of a new kind of coronavirus in 2019, the SARS-CoV-2, caused severe worldwide consequences and led to millions of deaths all over the world (1, 2).

The SARS-CoV-2 specific adaptive immune responses induced either by natural infection or vaccination play a crucial role in the control of the infection (3–7). In most acute viral infections, neutralizing antibodies increase rapidly after infection and persist for many years due to long-lasting plasma cells and memory B cells, playing vital roles in viral clearance and protection against viral diseases (8). However, it was shown that the antibody response triggered by coronaviruses in humans tends to wane over time (9). Currently, only few studies have investigated the dynamic change and the longevity of SARS-CoV-2 specific immune responses (10–13), although it is important to understand how natural reinfections, booster vaccination doses, different immunosuppressive drugs could influence the long-term immune response.

Patients with autoimmune rheumatic diseases have demonstrated heightened susceptibility to developing SARS-CoV-2 infection and hospitalization (14, 15), and as a consequence, they have been deemed a priority target group for the distribution of basic and booster vaccinations, too (16–18). Previously, we investigated the factors influencing the SARS-CoV-2 infection and vaccination-induced immune response in RA (19). In accordance with earlier investigations (20–22), we showed that following vaccination against COVID-19 (one or two vaccination doses) or SARS-CoV-2 infection, the induced anti-S antibodies decreased rapidly: approximately by 25% in each month, and we predicted that many RA patients would have sub-therapeutic antibody levels after 12 months. Additionally, based on our results, RA patients treated with tumor necrosis factor (TNF)-α-inhibitors, interleukin (IL)-6-inhibitor had reduced SARS-CoV-2-specific antibody response and anti-CD20 therapy inhibited both SARS-CoV-2-induced humoral and cellular immune responses (19).

Here, we studied the SARS-CoV-2-specific immunity longitudinally in rheumatoid arthritis patients and healthy controls.

## 2 Materials and methods

### 2.1 Patients

All 84 patients with RA included in this study were recruited from the rheumatology outpatient department of Semmelweis University (Buda Hospital of the Hospitaller Order of Saint John of God) in Budapest, Hungary. The study was conducted with the approval number IV/2021–1/2021/EKU from 19.10.2022 until 01.03.2023. Patients for the study were 18 years old or above who received COVID-19 vaccination and/or had previous confirmed SARS-CoV-2 infection and were diagnosed with RA according to the 2010 American College of Rheumatology/European League Against Rheumatism classification criteria (23). A control group consisting of 43 healthy individuals was also included in the study. These individuals were vaccinated against COVID-19 and/or had previous confirmed SARS-CoV-2 infection. Peripheral blood samples were collected to measure the SARS-CoV-2 induced humoral and cellular immune responses in all participants. Written, informed consent was obtained from all participants.

### 2.2 Evaluation of SARS-CoV-2-specific antibodies (Elecsys^®^ Anti-SARS-CoV-2 S assay, Roche; Euroimmun ELISA test)

The analysis of SARS-CoV-2-specific antibodies was performed using the Elecsys Anti-SARS-CoV-2 S immunoassay, developed by Roche. This assay was conducted on the Cobas e6000 instrument. The test is designed to detect antibodies specifically targeting the receptor binding domain (RBD) of the S protein of SARS-CoV-2 in human serum and plasma samples. The immunoassay uses a recombinant protein that represents the RBD of the S antigen in a double-antigen sandwich assay format. This format is particularly effective in detecting high-affinity antibodies (IgG, IgA, IgM) against SARS-CoV-2 in a quantitative manner. The method uses electrochemiluminescence for the quantitative determination of these antibodies. The limit of quantification for this assay is 0.4 U/ml, which means that results below this threshold are considered negative. Results equal to or above 0.8 U/ml are considered positive, as described in the test documentation (24).

The level of specific antibodies (IgG) against NCP from the SARS-CoV-2 virus in a blood sample was measured by Euroimmun ELISA test. It provides a semi-quantitative assessment, meaning it gives an approximate measurement rather than an exact value. The results are presented as a ratio, comparing the absorbance of the sample to a standard calibrator. If the ratio is below 0.8, it is considered negative for the presence of antibodies. Ratios equal to or above 1.1 are considered positive, indicating the presence of antibodies according to the test’s guidelines (25).

### 2.3 Measurement of IFN-γ T-cell responses (QuantiFERON (QIAGEN Group))

A small amount (1 ml) of heparinized whole blood was stimulated with specific antigens (S1, S2, RBD subdomains) that induce immune response in certain types of T-cells (CD4+ and CD4+CD8+ T-cells). In one test tube, additional specific peptides were added to cover various parts of the SARS-CoV-2 virus (spike, nucleocapsid, and M protein domains) to provoke a more comprehensive immune response from T-cells (Ag3). After incubation at 37°C for 16–24 hours, the levels of IFN-γ were measured using the ELISA QuantiFERON test. The measurement followed the instructions provided by the manufacturer. The manufacturer’s data sheet suggested that an IFN-γ level between 0.15 IU/ml and 0.2 IU/ml should be considered positive (26). However, in our study, we defined the positive cut-off as 0.15 IU/ml.

### 2.4 Statistical analysis

#### 2.4.1 Variable definitions

**Figure 1:**
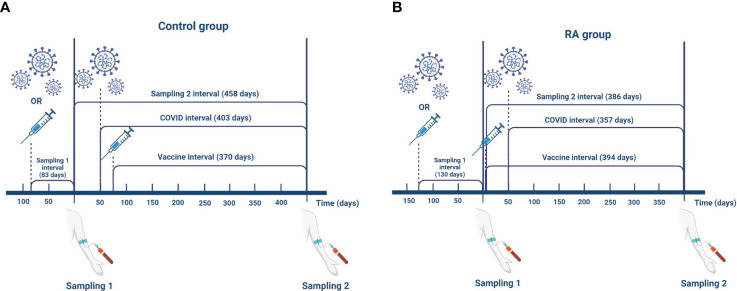
Definitions of the interval variables in the control group **(A)** and in the RA group **(B)**. The data shown in the figure represent median values. The time interval following the last event (SARS-CoV-2 infection or vaccination against COVID-19) until the first sampling is the “Sampling 1 interval” (control: 83 (37, 190) days; RA: 130 (81, 169) days). “Sampling 2 interval” is the time in days between Sampling 1 and Sampling 2 (control: 458 (410, 547) days; RA: 386 (311, 448) days). “Covid interval” is the time between confirmed case of SARS-CoV-2 infection by a positive NCP IgG test result and the Sampling 2 (control: 403 (267, 543) days; RA: 357 (245, 448) days). “Vaccine interval” is the time between additional vaccination against COVID-19 during the interim period and the Sampling 2 (control: 370 (330, 405) days; RA: 394 (342, 453) days). “Event interval” is defined as a time interval between vaccination against COVID-19 or SARS-CoV-2 infection during the interim period and sampling whichever is closer to the Sampling 2 (control: 194 (67, 327) days; RA: 321 (201, 378) days). If following the Sampling 1 none of these events happened then we set “Event interval” to equal to the “Sampling 2 interval”. This figure was created with BioRender.com.

The time interval in days following the last event (vaccination against COVID-19 or SARS-CoV-2 infection) until the first sampling is the “Sampling 1 interval”. “Sampling 2 interval” is the time in days between Sampling 1 and Sampling 2. “Covid interval” is the time in days between the confirmed case of SARS-CoV-2 infection by a positive NCP IgG test result and the Sampling 2. “Vaccine interval” is the time in days between additional vaccination against COVID-19 during the interim period and the Sampling 2. “Event interval” is defined as a time interval between vaccination against COVID-19 or SARS-CoV-2 infection during the interim period and sampling, whichever is closer to Sampling 2. If following Sampling 1, none of these events happened, then we set the “Event interval” to equal to the “Sampling 2 interval”. **Figure 1** illustrates these interval definitions and the median (IQR) elapsed time in days in both groups.

**Figure 2:**
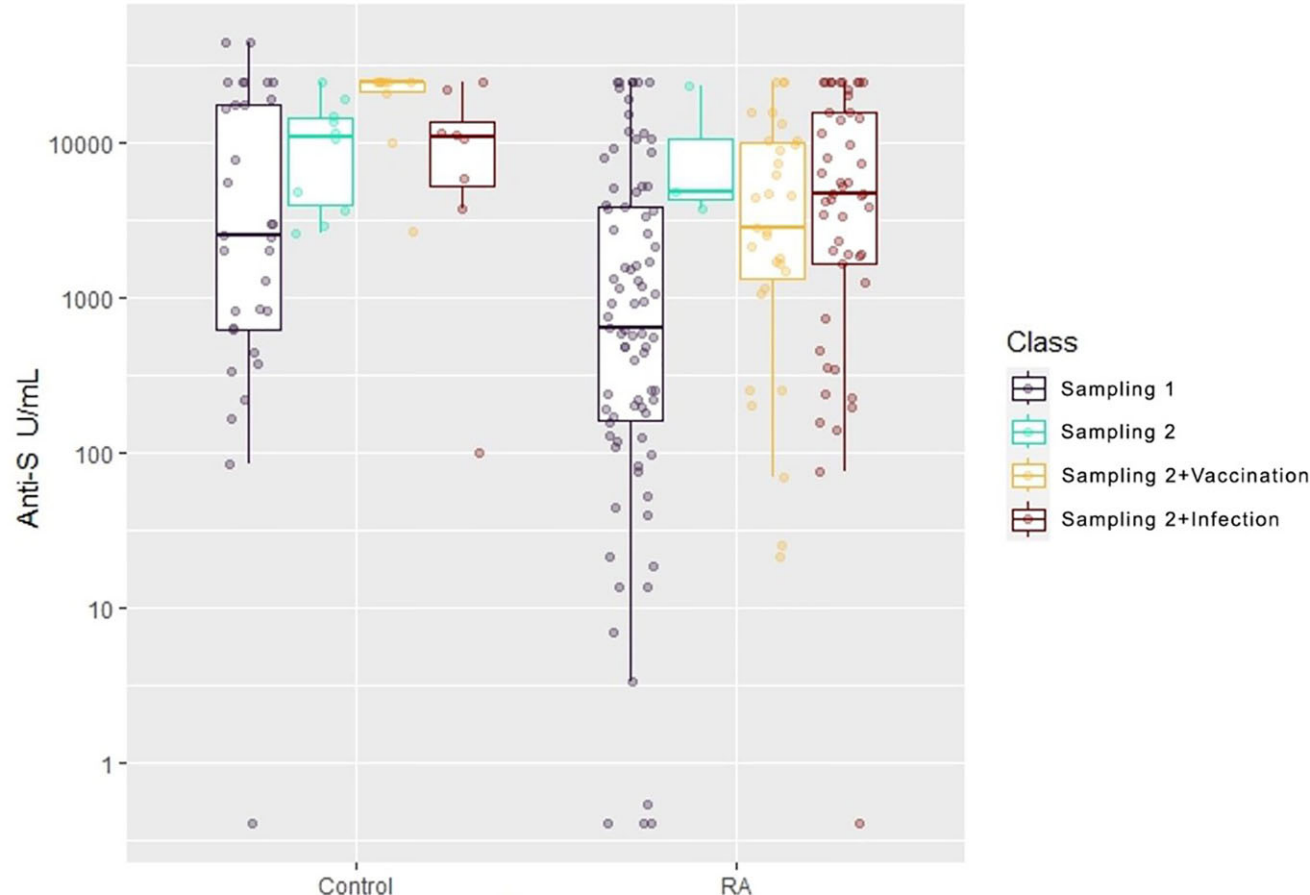
Boxplot of anti-S concentration by Group (healthy control or RA) and by subgroups. To ease the statistical and graphical analysis we classified observations into subgroups. Concentrations in subgroup “Sampling 1” were measured in the first period. Concentrations in subgroup “Sampling 2” were measured in the second period and neither vaccination against COVID-19 nor SARS-CoV-2 infection had been recorded during the interim period. Concentrations in subgroup “Sampling 2+Vaccination” were measured in the second period and during the interim period the participant received another vaccination dose against COVID-19. Concentrations in subgroup “Sampling 2+Infection” were measured in the second period and during the interim period there was a clinically documented SARS-CoV-2 infection. In a case of multiple events (vaccination against COVID-19 and SARS-CoV-2 infection) the classification is based on the type of the last event.

Concentrations in the “Sampling 1” subgroup were measured in the first period. Subgroup “Sampling 2” represents cases with no confirmed SARS-CoV-2 infection or additional vaccination against COVID-19 between the first and second measurements. Subgroup “Sampling 2+Vaccination” and “Sampling 2+Infection” consist of subjects who were either vaccinated against COVID-19 prior to the second measurement or had confirmed SARS-CoV-2 infection before the second measurement. It is important to note that the term “prior to measurement” indicates that latest vaccination against COVID-19 or latest SARS-CoV-2 infection was the most recent event that triggered the SARS-CoV-2-specific immune response. For instance, if a subject received a COVID-19 vaccine first and later had a SARS-CoV-2 infection, their sample would still be classified as “Sampling 2+Infection” (**Figure 2**).

It should be noted that data from the first period could not be split this way. Before taking the first sample, every participant experienced at least one event of SARS-CoV-2 infection or vaccination against COVID-19. Furthermore, in the first period, vaccines with varying efficacies against COVID-19 were administered (BNT162b2, mRNA-1273, ChAdOx1s, Gam-COVID, BBIBP-CorV) whereas, in the interim period, only RNA-based vaccines such as BNT162b2 vaccine and mRNA-1273 vaccine were used.

The data shown in **Figure 2** underwent analysis using stepwise linear regression. The stepwise algorithm identified and eliminated variables such as sex, group (healthy control or RA), MTX use, and low dose of corticosteroid therapy as not statistically significant.

#### 2.4.2 Descriptive statistics and simple hypothesis testing

Continuous variables are presented as median (IQR) and compared using the Wilcoxon rank-sum test or unpaired t-test. Categorical variables are presented as n (percent) and compared using the chi-square test. The reported p-values are the nominal p-values without correction for multiple hypothesis testing.

#### 2.4.3 Regression modeling strategy

Linear regression was used to model anti-S dependence on external factors. Based on our previous results, the initial linear regression model contained the following variables: Sex, Age, Group (healthy or RA), “Event interval”, the nature of the last event (vaccination against COVID-19 or SARS-CoV-2 infection), and Period. Period is a categorical variable that can take two values, demonstrating if a measured value comes from the first (Sampling 1) or follow-up period (Sampling 2).

Anti-S concentrations were highly right-skewed. Therefore, concentrations were logarithmically transformed before entering into regressions models. Anti-S half-life was estimated assuming using the log (0.5)/” Event interval” formula where “Event interval” is the estimated regression coefficient of the “Event interval” variable.

Additionally, only RA patients received targeted therapy. Therefore, from a statistical perspective, variables such as group (healthy control or RA), age, and targeted therapy are interdependent. In such cases, it is recommended to look beyond individual p-values and consider the overall fit of the model. Accordingly, we fit the two regression models. Model 1 included SARS-CoV-2 infection, vaccination against COVID-19, “Event interval” and “Sampling time point” variables, while Model 2 contained the variables mentioned before plus the group, age, and targeted therapy variables. The two models were compared with the F test. The corresponding F is highly significant (F= 7.34, df= 208, 211, p= 0.0001059), showing that the patient group differs considerably from the healthy volunteer group.

The correlation between anti-S level and CW4–8 T-cell response was evaluated by non-parametric Spearman’s rank test. A Spearman’s rho >0.7 indicated a high correlation, 0.7 > rho > 0.5 indicated a moderate one, and rho <0.5 indicated a low correlation. Two-tailed p values were considered significant if <0.05.

#### 2.4.4 Software

Data were analyzed using R (27), and tables and figures were prepared with the gtsummary (28) and ggplot2 (29).

## 3 Results

### 3.1 Study population

84 RA patients and 43 healthy controls were included in our longitudinal study (**Table 1**). The activity of RA was assessed by examining the patient clinically using a scoring system called the Disease Activity Score 28-CRP (DAS28-CRP). The median DAS28-CRP score was 2.6. In this scoring system, a DAS28-CRP value above 5.1 indicates high disease activity, values between 3.2 and 5.1 indicate moderate activity, values between 2.6 and 3.2 indicate low activity and values between 0 and less than 2.6 indicate remission of the disease (30). In the RA group, 63 (75%) patients were treated with methotrexate (MTX) as monotherapy or in combination, and 20 patients (24%) received a low dose of corticosteroid therapy (all of them below 7.5 mg/day prednisone or equivalent). The majority of RA patients (N=75; 89%) were treated with targeted therapy (TNF-α inhibitors, IL-6-inhibitor, rituximab, JAK inhibitors). In the baseline study (19), RA patients received COVID-19 vaccination 3.5 ± 0.87 months (SD/SEM, n=4) after rituximab treatment. Of the 84 RA patients in the follow-up study, only one was treated with rituximab and received the COVID-19 vaccine four months after the rituximab dose. In the first phase, SARS-CoV-2 infection was confirmed with a positive PCR test. In the second phase, if a SARS-CoV-2 infection occurred, the role of SARS-CoV-2 was confirmed by detecting IgG antibodies against NCP as a response marker. During the interim period, only RNA-based vaccines, such as the BNT162b2 vaccine and the mRNA-1273 vaccine, were administered. **Figure 1** reveals that during the follow-up phase, the duration between the events that triggered the SARS-CoV-2-specific immune response and the time of sampling was considerably extended compared to the first period.

**Table 1:** Descriptive characteristics of the study population.

Characteristic	Control, N =43	RA, N = 84	p-value
**Sex**			<0.001
Male	22 (51%)	13 (15%)	
Female	21 (49%)	71 (85%)	
**Age**	42 (33, 53)	63 (52, 69)	<0.001
Therapy status			
MTX	Not applicable	63 (75%)	
Glucocorticoid	Not applicable	20 (24%)	
Targeted therapy^1^	Not applicable	75 (89%)	
SARS-CoV-2 infection status			
SARS-Cov-2 positivity during Sampling 1 Positive PCR test^2^	26 (60%)	38 (45%)	0.10
SARS-Cov-2 positivity during Sampling 2 Positive NCP IgG^3^ (≥ 1.1)	16 (57%)	29 (35%)	0.039
COVID-vaccination status^4^			
4 doses of COVID-vaccination	3 (11%)	12 (14%)	
3 doses of COVID-vaccination	21 (75%)	57 (68%)	
2 doses of COVID-vaccination	3 (11%)	13 (15%)	
1 dose of COVID-vaccination	0	1 (1%)	
Unvaccinated	1 (4%)	1 (1%)	

^1^TNF-α inhibitors, IL-6-inhibitor, rituximab, JAK inhibitors; **^2^** SARS-CoV-2 infection was confirmed with a positive PCR test in the first part of the study. **^3^** In the second test part, we confirmed the SARS-CoV-2 infection with a positive NCP IgG test result. **^4^** BNT162b2, mRNA-1273, ChAdOx1s, Gam-COVID, BBIBP-CorV COVID-vaccines were administrated. Continuous variables are presented as median (IQR) and compared using the Wilcoxon rank-sum test, and categorical variables are presented as n (%) and compared using the chi-square test. RA, rheumatoid arthritis; NCP, nucleocapsid protein; MTX, methotrexate.

### 3.2 Anti-SARS-CoV-2 antibody response

**Figure 3:**
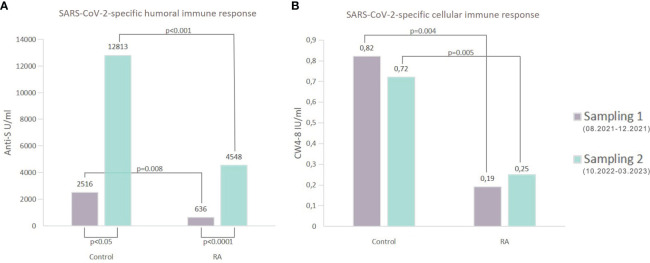
Comparative analysis of the SARS-CoV-2-specific immune responses. **(A)** shows the median of anti-S concentrations in both groups. Regardless of the Sampling time point (Sampling 1 or Sampling 2), patients with RA showed statistically significantly lower levels of anti-S antibodies when compared to the healthy individuals (Sampling 1: 636 U/ml (RA) vs 2516 U/ml (control) p=0.008; Sampling 2: 4548 U/ml (RA) vs 12813 U/ml (control) p<0.001). Furthermore, compared to the first measurements in the second period, there was a large increase in the SARS-CoV-2-specific humoral immune response. These differences were significant for both groups (p<0.05 for healthy volunteers, p<0.0001 for RA patients, rank-sum test). **(B)** shows the median of whole COVID virus-stimulated COVID-specific CD4+ and CD8+ T-cell response (CW4–8) in healthy controls and patients diagnosed with RA. After considering measurements from both periods (Sampling 1 and Sampling 2), it was observed that RA patients had a diminished CW4–8 T-cell response in comparison to healthy controls.

**Figure 4:**
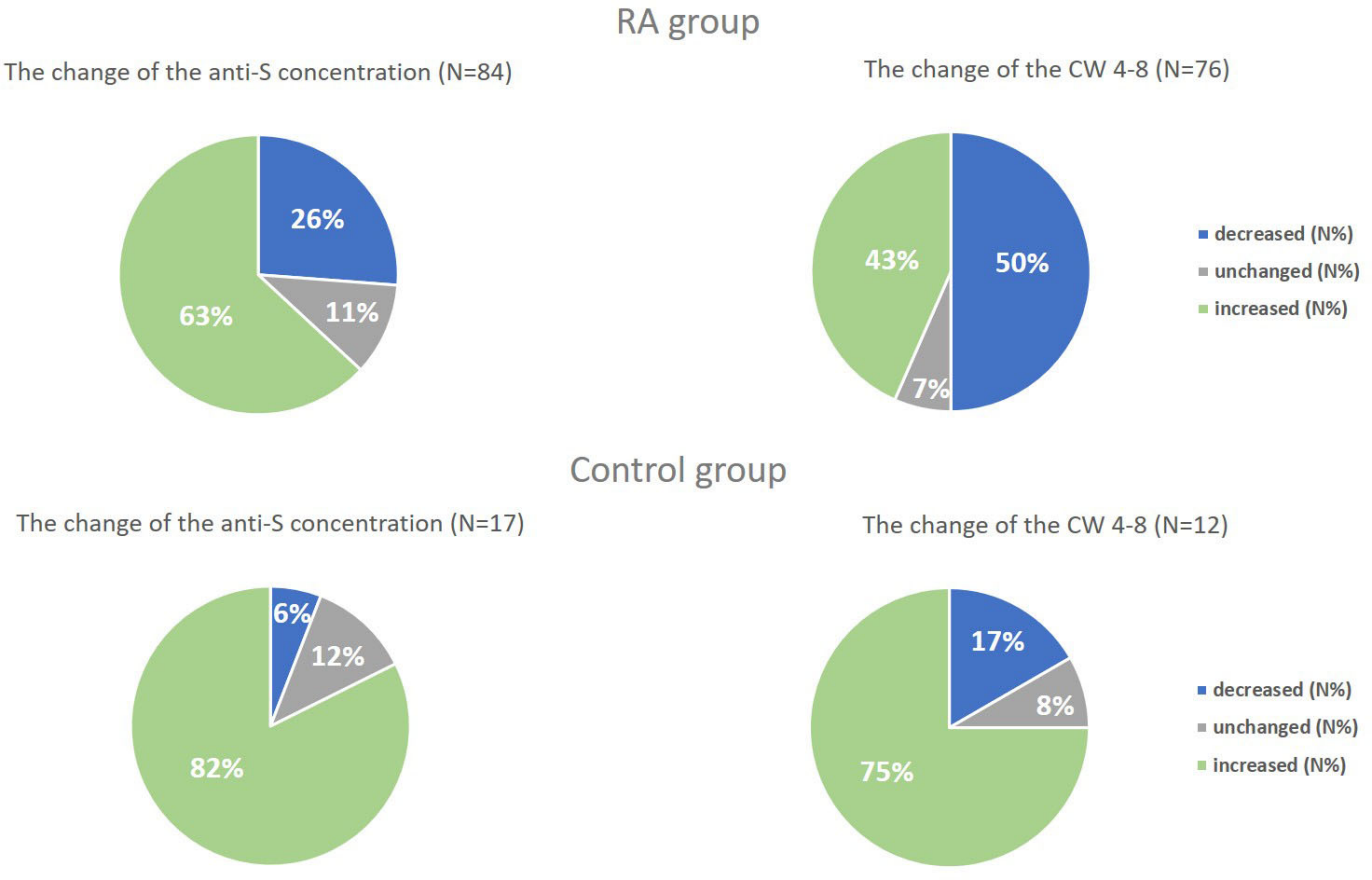
The change of the anti-S concentration and the whole COVID virus-stimulated COVID-specific CD4+ and CD8+ T-cell response (CW 4–8) in both study groups. We compared the results of Sampling 1 and Sampling 2 and we defined a change of at least 10% decrease or increase, “unchanged” means the rate of the change is less than 10%.

Regardless of the period, patients with RA had lower anti-S levels compared to healthy controls (p<0.01 for healthy controls, p<0.001 for RA patients, **Supplementary Table 1**). The median (IQR) concentration of the anti-S antibodies was 4548 U/ml (1567, 13949) in the RA group and 12813 U/ml (5632, 25000) in the healthy group based on the follow-up results (**Supplementary Table 1**). Additionally, there was a massive increase in the SARS-CoV-2-specific humoral immune response in each study population during the follow-up period (p<0.05 for healthy volunteers, p<0.0001 for RA patients, rank-sum test) (**Figure 3A**). 63% (53/84) of RA patients and 82% (14/17) of healthy individuals had increased SARS-CoV-2-specific humoral immune response compared to the baseline results (**Figure 4**). The median values of the anti-S levels increased sevenfold in the RA group and fivefold in the control group compared to the baseline results.

**Table 2** shows that the “Sampling time point” effect, with an estimate of 9.34 (CI95%: 4.58–19.00, p<0.001), indicates that the anti-S concentrations in the second sampling period were several times higher than those in the first period after adjusting for covariates. Furthermore, the “Sampling time point” effect is multiplied by a factor of 2.72 (CI 95%: 1.25–5.95, p < 0.05) if a study subject received a vaccination against COVID-19 during the interim period (**Figure 2**). Additionally, individuals who had a confirmed SARS-CoV-2 infection during the interim period had anti-S levels that were 2.1 times higher (CI 95%: 1.31–3.37) compared to those who did not have SARS-CoV-2 infection (p<0.01) (**Figure 2**). However, the effects of these two events gradually diminish over time, with an estimated half-life of 176.8 days (CI 95%: 51.9–301.8 days, p<0.01). But at the same time, the multiplying factor targeted therapy is 0.48 (CI 95% 0.25–0.94, p<0.05), meaning that compared to matched patients, the anti-S levels are 52% lower. Moreover, anti-S concentrations decrease with age, with each 20-year difference corresponding to a decrease of approximately 45.6% (calculated as 100(1–0.9720)).

**Table 2:** The main factors influencing anti-S antibody levels in each study population.

Characteristic	exp (Beta) (95% CI)	p-value
Vaccination against COVID-19	2.72 (1.25 to 5.95)	0.013
SARS-CoV-2 infection	2.10 (1.17 to 3.76)	0.014
Sampling time point	9.34 (4.58 to 19.0)	<0.001
Targeted therapy	0.48 (0.25 to 0.94)	0.033
Age	0.97 (0.94 to 0.99)	0.004

Sampling time point: Sampling 1 or Sampling 2. For discrete variables, the regression parameters are multiplication factors that show how many times the anti-S concentration increases (decreases) in the presence versus absence of the given factor. For age, the parameter is the yearly percental change. CI, Confidence Interval.

Based on the COVID-19 vaccination (three doses) history of the participants, 77% (44/57) of RA patients and 76% (16/21) of healthy controls received homologous COVID-19 vaccinations. In contrast, 23% (13/57) of RA patients and 24% (5/21) of healthy controls received heterologous vaccine doses. For those who received homologous vaccinations, the median (IQR) concentration of anti-S antibodies was 6362 U/ml (1989, 15696) in the RA group and 14163 U/ml (5632, 24842) in the healthy group. For those who received heterologous vaccinations, the median (IQR) concentration of anti-S antibodies was 4625 U/ml (1664, 13532) in the RA group and 21986 U/ml (19524, 25000) in the healthy group. Based on our results there was no significant difference between homologous (9382 U/ml (2582, 21347)) and heterologous (9588 (2858, 21604)) vaccination groups, if we calculate the median (IQR) concentration for the whole study population.

**Figure 5:**
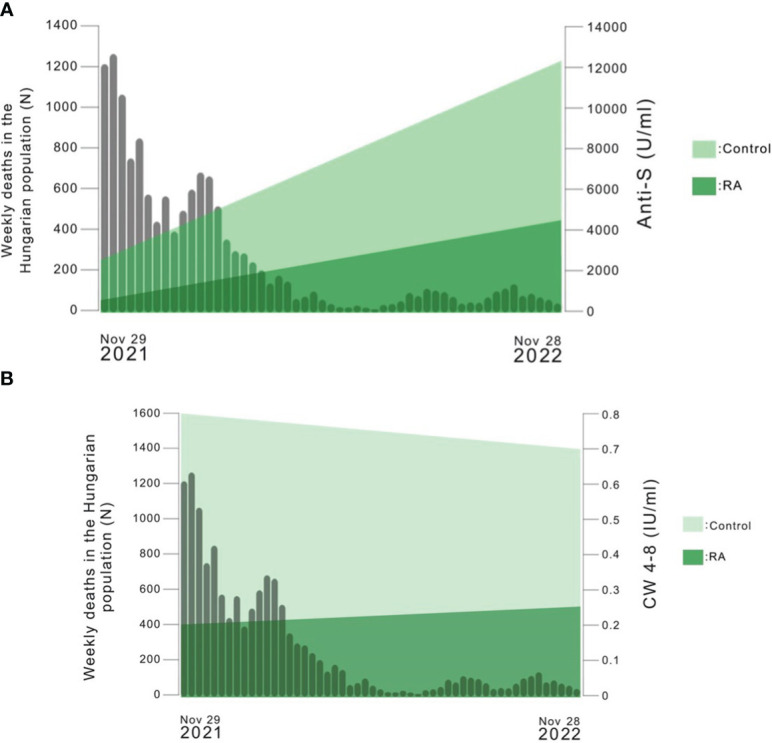
**(A)** The number of weekly deaths caused by SARS-CoV-2 infection in the Hungarian population versus the median of anti-S levels over time in each study population (https://covid19.who.int/region/euro/country/hu). The median values of the results of Sampling 1 and Sampling 2 were connected with a line in the Figure. **(B)** The number of weekly deaths caused by SARS-CoV-2 infection in the Hungarian population versus the median of whole COVID virus-stimulated COVID-specific CD4+ and CD8+ T-cell response (CW 4–8) over time in each study population (https://covid19.who.int/region/euro/country/hu). The median values of the results of Sampling 1 and Sampling 2 were connected with a line in the Figure.

**Figure 5A** shows the number of weekly deaths caused by SARS-CoV-2 infection in Hungary between 29.11.2021 and 28.11.2022 versus the median of anti-S levels over time in each study population. Based on these data, the number of weekly deaths caused by SARS-CoV-2 infection in Hungary and the median of anti-S levels changed in opposite ways over time. The heightened level of anti-S antibodies might explain the decline in the number of weekly deaths caused by SARS-CoV-2 infection.

### 3.3 IFN-γ T-cell response

By investigating the SARS-CoV-2-specific T-cell response, we were able to study how it changes when stimulated by three specific antigens (I: spike antigen stimulated COVID-specific CD4+ (CD4); II: spike antigen stimulated COVID-specific CD4+ and CD8+ (CD4–8) and III: whole COVID virus-stimulated COVID-specific CD4+ and CD8+ (CW4–8) T-cell responses). We further analyzed the CW4–8 T-cell response because it seemed to be the most informative marker.

Based on both measurements (Sampling 1, Sampling 2), RA patients had reduced CW4–8 T-cell response compared to healthy controls (p<0.01 for healthy controls, p<0.001 for RA patients, **Figure 3B**). We identified a median IFN-γ level (reflecting CW4–8 T-cell response) of 0.72 IU/ml (0.52, 1.25) in healthy controls; however, the magnitude of the RA patients’ response was 0.25 IU/ml (0.05, 1.28) (**Supplementary Table 1**). 43% (33/76) of RA patients and 75% (9/12) of healthy individuals had increased CW4–8 T-cell response compared to the baseline results (**Figure 4**). A significant, low correlation was observed between the anti-S antibody level and the CW4–8 T-cell response in each study population (r=0.3891, p<0.0001).

**Supplementary Figure 1** shows markers of SARS-CoV-2-specific T-cell response. No apparent trend or pattern is observable. Indeed, a similar linear regression modeling strategy applied for anti-S did not yield any significant effect, except CW4–8, where SARS-CoV-2 infection (but not vaccination against COVID-19) increased the CW4–8 response to 2.09-fold (95% CI: 1.30 - 3.36, p<0.01) in RA patients.

**Figure 5B** shows the number of weekly deaths caused by SARS-CoV-2 infection in Hungary between 29.11.2021 and 28.11.2022 versus the median of CW4–8 T-cell response over time in each study population. Based on these data, the number of weekly deaths caused by SARS-CoV-2 infection in Hungary decreased over time, however, the level of CW4–8 T-cell response had not changed substantially in the study groups.

## 4 Discussion

In this longitudinal study, we followed a cohort of rheumatoid arthritis patients and healthy individuals to assess their SARS-CoV-2-specific humoral and cellular immune response over one year.

Neutralizing antibodies are specific to viral epitopes that are predominantly in the spike protein and they play an important protective role by blocking the virus from entering the host cells, which limits infection (31). However, studies revealed that the level of neutralizing antibodies decreases over time following either SARS-CoV-2 infection or vaccination against COVID-19, thereby increasing the chance of potential reinfections (32–34). The SARS-CoV-2 virus has been notable for its extensive array of genomic variations observed since the beginning of the pandemic (35). These variations have led to significant changes across all structural proteins, driven by natural selection, resulting in the emergence of variants with heightened abilities in transmission and replication (36). From November 2021, the majority of infections were caused by the omicron (B.1.1.529) coronavirus variant, which caused the most infections (37) and since then, it has undergone numerous mutations (38). One reason for the rapid spread of mutants is the infection spread by mild and asymptomatic virus carriers (39). It has remained unclear, how asymptomatic SARS-CoV-2 infections could affect the dynamic and the longevity of the SARS-CoV-2-specific immunity, especially in patients receiving immunosuppressive therapy.

Previous studies (15, 40–42) announced that immunocompromised patients, including RA patients, have a higher prevalence of SARS-CoV-2 infection and have a higher mortality risk of COVID-19 compared to the general population. We confirmed the results of our first report (6) and earlier findings (18–23), patients diagnosed with RA had substantially lower anti-S levels even after more than a yearlong interim period compared to the healthy control group (p<0.01 for healthy controls, p<0.001 for RA patients). We also evaluated the CW4–8 T-cell response during the follow-up period, and similarly to other research groups (43–46), we found that patients diagnosed with RA showed a diminished CW4–8 T-cell response in comparison to the healthy control group (p<0.01 for healthy controls, p<0.001 for RA patients). It was found that patients with immune-mediated inflammatory diseases have a lower and less long-lasting SARS-CoV-2-specific immune response than healthy individuals, so earlier booster doses or more frequent re-doses, or both, are recommended in order to achieve an adequate immune response in this population (47). Furthermore, it was shown that booster vaccination doses against COVID-19 enhances the SARS-CoV-2-specific immune response of immunocompromised patients (48) and increases the vaccine effectiveness against COVID-19-related death in both immunocompromised patients and healthy individuals (42). Although glucocorticoid therapy may affect the vaccination-induced immune response, in line with the appropriate ACR guidance (43) glucocorticoid therapy at the lowest effective dose was continued, because (not delaying the COVID-19 vaccination) uncontrolled disease activity poses a greater risk than the potential modification of vaccine response due to glucocorticoid use.

**Figure 6:**
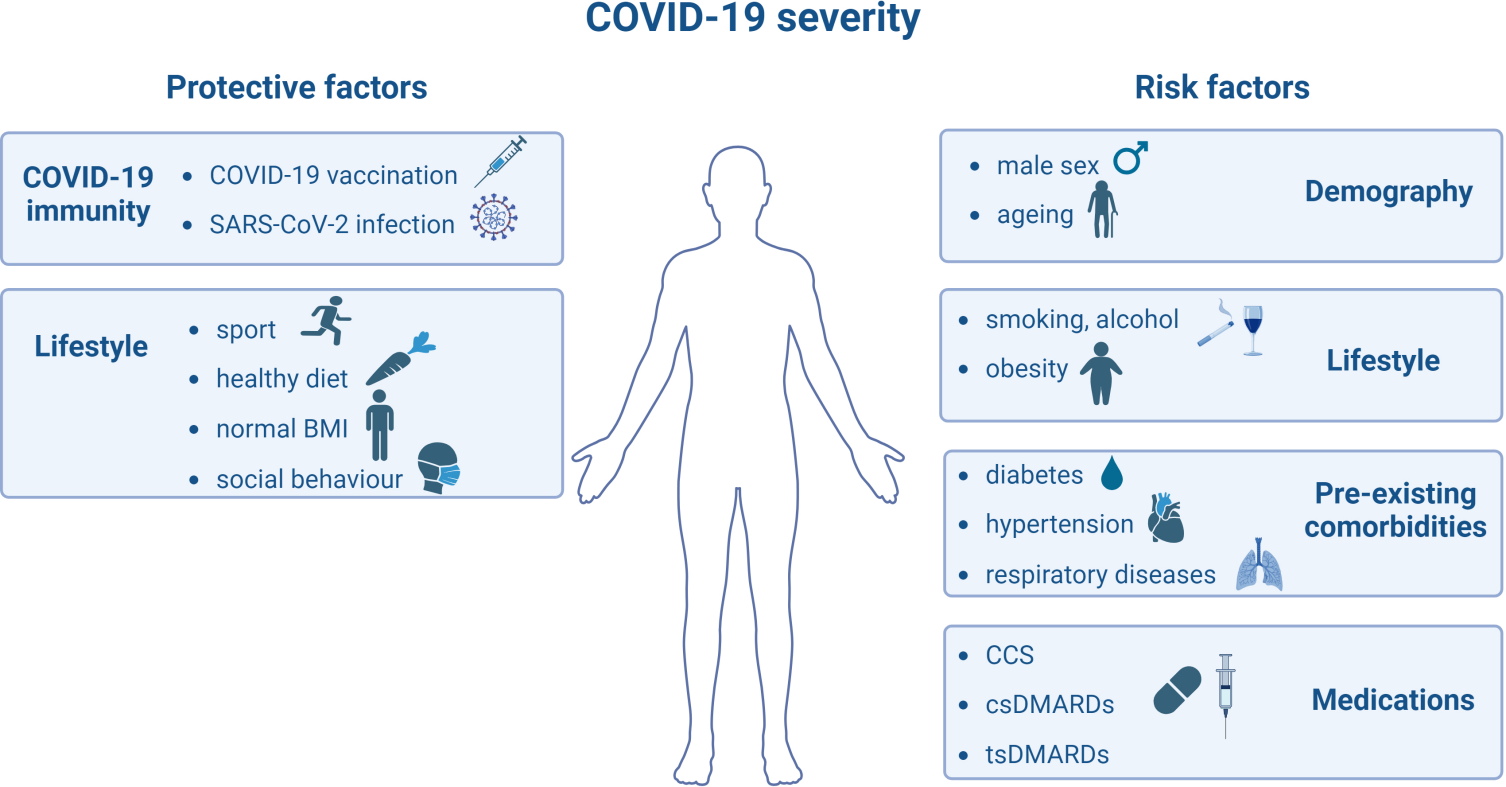
Protective and risk factors of COVID-19 severity. BMI: Body mass index. CCS: corticosteroids. csDMARDs: conventional synthetic disease-modifying antirheumatic drugs. tsDMARDs: targeted synthetic disease-modifying antirheumatic drugs. This figure was created with BioRender.com.

In contrast with other studies (32–34), throughout the follow-up period, both RA patients and healthy volunteers showed several times higher anti-S concentrations, regardless of whether they were vaccinated against COVID-19 or had previous SARS-CoV-2 infection during the time between the first and second samplings (as shown in **Figures 2**, **3** and **Supplementary Table 1**). The same laboratory methods were used in order to measure the level of SARS-CoV-2-specific humoral and cellular immune responses at the two Sampling time points. These findings were somewhat unexpected, as based on our previous analysis (19), we expected lower rather than substantially higher anti-S levels under these conditions. Additionally, according to **Figures 5A, B** as the number of weekly deaths caused by SARS-CoV-2 infection in the Hungarian population decreased, the median anti-S concentrations increased and CW4–8 T-cell response remained relatively stable in both study populations. Our results confirmed a previously described fact that anti-S antibody concentrations have been shown to correlate with protection against COVID-19 (46, 49, 50). Moreover, earlier studies marked that retained CD4+ and CD8+ T-cell responses were associated with reduced COVID-19 severity (44–46) and T-cell immunity to the SARS-CoV-2 may help to compensate the absence of neutralizing antibodies in preventing or limiting severe COVID-19 (33, 49). In addition, there are many protective and risk factors of COVID-19 severity (50) as shown in **Figure 6**. The importance of the CW4–8 T-cell response was confirmed by our previous work (51), where we assessed the potential predictive value of CW4–8 T-cell response and SARS-CoV-2-specific humoral immune response for survival in critically ill COVID-19 patients requiring venovenous extracorporeal membrane oxygenation (VV-ECMO). We found that CW4–8 T-cell response was significantly higher among survivors compared to the deceased patients.

This is an observational study with its inherent limitations. However, because of these limitations, we cannot say for sure whether the reduced humoral response in the RA group was due to the disease itself, the targeted therapy, or simply the fact that the patients were older than the healthy volunteers. Statistical analysis suggests that the last two (treatment with targeted therapy and age, **Table 2**) better explain the difference between the healthy volunteer and patient group than the disease itself, which aligns with our previous results (19). There is a significant difference between the healthy and the RA groups in terms of age and gender. However, the gender effect on the anti-S response is negligible, and the significant age effect was adjusted for all other variables. The reported p-values have been “cleaned” of the age effect.

In summary, the increased SARS-CoV-2-specific humoral immune response, the nearly permanent CW4–8 T-cell response, and possibly the simultaneous presence of various SARS-CoV-2 variants in the population potentially resulting in mild or symptom-free cases of COVID-19, may contribute to the low number of severe COVID-19 cases in the whole population.

## Data availability statement

The original contributions presented in the study are included in the article. Further inquiries can be directed to the corresponding author.

## Ethics statement

The studies involving humans were approved by National Public Health Center, Hungary. IV/2021-1/2021/EKU. The studies were conducted in accordance with the local legislation and institutional requirements. The participants provided their written informed consent to participate in this study.

## Author contributions

DN: Writing – original draft, Writing – review & editing. HV: Writing – review & editing. LT: Writing – review & editing. ZU: Writing – review & editing. DB: Writing – review & editing. ZS: Writing – review & editing. BR: Writing – review & editing. LG: Writing – review & editing. BM: Writing – original draft, Writing – review & editing. GN: Writing – original draft, Writing – review & editing.
