# Supplementary material for: Escalating SARS-CoV-2 specific humoral immune response in rheumatoid arthritis patients and healthy controls
Source: Front Immunol. 2024 Jun 7;15:1397052. doi: 10.3389/fimmu.2024.1397052 (PMC11190160; doi:10.3389/fimmu.2024.1397052)

Supplementary Material

# Supplementary Table

**Supplementary Table 1.** Comparative analysis of the immune responses. Continuous variables are presented as median (IQR) and compared using the Wilcoxon rank-sum test. RA: rheumatoid arthritis; anti-S: Spike protein antibody.

| Characteristic | Control, N = 43 | RA, N = 84 | p-value |
| --- | --- | --- | --- |
| Baseline anti-S response (U/ml) | 2,516 (632, 17,898)  *n=34* | 636 (162, 3,932)  *n=84* | 0.008 |
| Follow-up anti-S response (U/ml) | 12,813 (5,632, 25,000)  *n=28* | 4,548 (1,567, 13,949)  *n=84* | <0.001 |
| Baseline spike antigen stimulating COVID-specific CD4+ T-cell response (CD4) (IU/ml) | 0.37 (0.16, 0.99)  *n=34* | 0.12 (0.02, 0.52)  *n=84* | 0.010 |
| Follow-up spike antigen stimulating COVID-specific CD4+ T-cell response (CD4) (IU/ml) | 0.38 (0.27, 0.97)  *n=28* | 0.06 (0.01, 0.43)  *n=76* | <0.001 |
| Baseline spike antigen stimulating COVID-specific CD4+ and CD8+ T-cell response (CD4-8) (IU/ml) | 0.59 (0.33, 1.31)  *n=34* | 0.13 (0.03, 0.80)  *n=84* | <0.001 |
| Follow-up spike antigen stimulating COVID-specific CD4+ and CD8+ T-cell response (CD4-8) (IU/ml) | 0.45 (0.32, 0.76)  *n=28* | 0.12 (0.01, 0.82)  *n=76* | 0.002 |
| Baseline whole COVID virus-stimulated COVID-specific CD4+ and CD8+ T-cell response (CW4-8) (IU/ml) | 0.82 (0.31, 1.61)  *n=34* | 0.19 (0.04, 1.02)  *n=84* | 0.004 |
| Follow-up whole COVID virus-stimulated COVID-specific CD4+ and CD8+ T-cell response (CW4-8) (IU/ml) | 0.72 (0.52, 1.25)  *n=28* | 0.25 (0.05, 1.28)  *n=76* | 0.005 |

# Supplementary Figure

**Supplementary Figure 1.** Boxplots of markers of SARS-CoV-2-specific cellular immune response by Group and subgroupes. The caption to Figure 4 provides the definition of subgroups. A: spike antigen stimulating COVID-specific CD4+ T-cell response (CD4); B: spike antigen stimulating COVID-specific CD4+ and CD8+ T-cell response (CD4-8); C: whole COVID virus-stimulated COVID-specific CD4+ and CD8+ T-cell response (CW4-8).


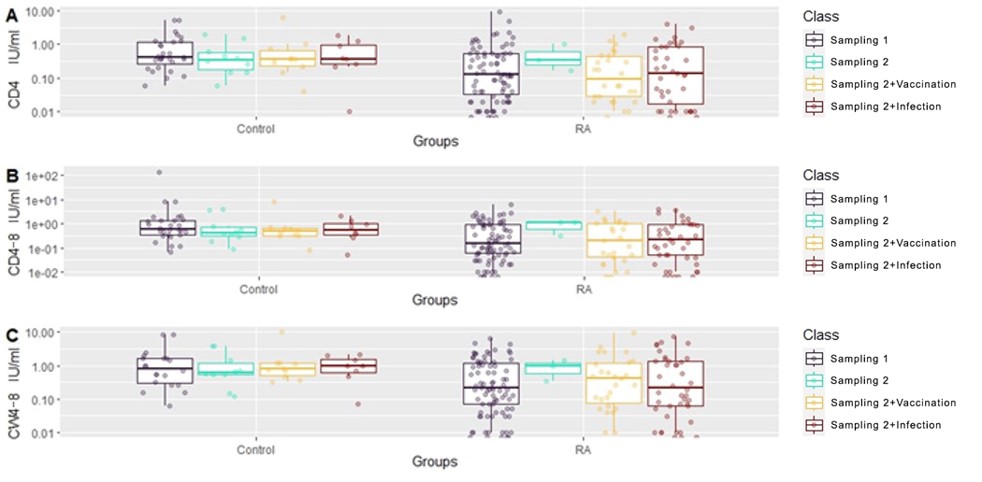

Supplement: Supplementary file 1 [file DataSheet_1.docx]
